# Midwife-led birthing centres in four countries: a case study

**DOI:** 10.1186/s12913-023-10125-2

**Published:** 2023-10-17

**Authors:** Oliva Bazirete, Kirsty Hughes, Sofia Castro Lopes, Sabera Turkmani, Abu Sayeed Abdullah, Tasleem Ayaz, Sheila E. Clow, Joshua Epuitai, Abdul Halim, Zainab Khawaja, Scovia Nalugo Mbalinda, Karin Minnie, Rose Chalo Nabirye, Razia Naveed, Faith Nawagi, Fazlur Rahman, Saad Ibrahim Rasheed, Hania Rehman, Andrea Nove, Mandy Forrester, Shree Mandke, Sally Pairman, Caroline S. E. Homer

**Affiliations:** 1 College of Medicine and Health, Sciences, University of Rwanda, Kigali, Rwanda; 2 Novametrics Ltd, Duffield, UK; 3 Independent Consultant, Cape Town, South Africa; 4 Burnet Institute, Melbourne, Australia; 5 Centre for Injury Prevention and Research, Bangladesh (CIPRB), Dhaka, Bangladesh; 6 Independent Consultant, Islamabad, Pakistan; 7 University of Cape Town, Rondebosch, South Africa; 8 Busitema University, Tororo, Uganda; 9 Research & Development Solutions, Islamabad, Pakistan; 10 Makerere University, Kampala, Uganda; 11 University of the Western Cape, Cape Town, South Africa; 12 International Confederation of Midwives, The Hague, Netherlands

**Keywords:** Midwife led birthing centre, Midwifery, Childbirth, Low and middle income countries, Bangladesh, Pakistan, South Africa, Uganda

## Abstract

**Background:**

Midwives are essential providers of primary health care and can play a major role in the provision of health care that can save lives and improve sexual, reproductive, maternal, newborn and adolescent health outcomes. One way for midwives to deliver care is through midwife-led birth centres (MLBCs). Most of the evidence on MLBCs is from high-income countries but the opportunity for impact of MLBCs in low- and middle-income countries (LMICs) could be significant as this is where most maternal and newborn deaths occur. The aim of this study is to explore MLBCs in four low-to-middle income countries, specifically to understand what is needed for a successful MLBC.

**Methods:**

A descriptive case study design was employed in 4 sites in each of four countries: Bangladesh, Pakistan, South Africa and Uganda. We used an Appreciative Inquiry approach, informed by a network of care framework. Key informant interviews were conducted with 77 MLBC clients and 33 health service leaders and senior policymakers. Fifteen focus group discussions were used to collect data from 100 midwives and other MLBC staff.

**Results:**

Key enablers to a successful MLBC were: (i) having an effective financing model (ii) providing quality midwifery care that is recognised by the community (iii) having interdisciplinary and interfacility collaboration, coordination and functional referral systems, and (iv) ensuring supportive and enabling leadership and governance at all levels.

**Conclusion:**

The findings of this study have significant implications for improving maternal and neonatal health outcomes, strengthening healthcare systems, and promoting the role of midwives in LMICs. Understanding factors for success can contribute to inform policies and decision making as well as design tailored maternal and newborn health programmes that can more effectively support midwives and respond to population needs. At an international level, it can contribute to shape guidelines and strengthen the midwifery profession in different settings.

**Supplementary Information:**

The online version contains supplementary material available at 10.1186/s12913-023-10125-2.

## Background

Access to quality sexual, reproductive, maternal, newborn and adolescent health care is essential for countries to achieve universal health coverage (UHC). Midwives are key in this endeavour. Midwives have the potential to impact maternal and newborn health outcomes, if they are adequately educated, deployed and supported in an enabling work environment. Universal access to midwife-delivered interventions could prevent two-thirds of the world’s maternal and newborn deaths and stillbirths [1]. 

Midwife-led birthing centres (MLBCs) are an option in many countries [2]. MLBCs are usually provided at a primary care level, have a dedicated space providing childbirth care where midwives are the lead professionals and have arrangements for consultation and referral to secondary and tertiary-level services if needed. Some MLBCs are close to, or an integral part of, a maternity or general hospital (called “alongside” or “on-site” centres) while others are situated apart from hospitals (called “stand-alone” or “freestanding” centres).

MLBCs that are appropriately integrated into the health system have been found to improve maternal and neonatal outcomes by providing woman-centred, high-quality, cost-effective, and safe care [3–8]. A recent Cochrane review examined 15 trials with 17,674 women from high-income countries and found that midwife-led continuity of care offers various favourable outcomes for women when compared to other care models [9].  

Most of the evidence on MLBCs is from high-income countries, but the impact of MLBCs in low- and middle-income countries (LMICs) could be significant as this is where the vast majority of maternal and newborn mortality and morbidity occurs [10]. However, we cannot assume that research findings from HICs can be generalised to other settings. For example, MLBC clients in HICs tend to belong to more wealthy demographics, [3] whereas in many low- and middle-income countries (LMICs), MLBCs primarily cater to clients from disadvantaged and marginalized communities [11].  The weak referral systems evident in many LMICs may compromise the safety of women and newborns who use MLBCs, especially if the MLBC is a freestanding one (the most prevalent type in LMICs [11]), whereas in HICs the evidence indicates better outcomes in freestanding MLBCs [3].  Further, in low-income nations, MLBC services are predominantly offered by private, not-for-profit entities. Consequently, the accessibility of care for clients from underprivileged and marginalized backgrounds heavily relies on sustained support from these sources [11].  

Our previous work has shown that MLBCs exist in at least 24 LMICs [11]. Much of the evidence about these MLBCs was collected in a survey and there was limited information in the peer reviewed literature, especially understanding how the MLBCs were successful. The aim of this study therefore, was to explore MLBCs in four low- and middle income countries, specifically to understand what is needed for a successful MLBC.

## Methods

### Study design

A descriptive study with four case study countries was undertaken using an Appreciative Inquiry approach [12]. Appreciative Inquiry is a participatory technique designed to initiate a positive conversation about experiences, needs, and proposed solutions [13, 14]. The study was guided by a networks of care (NOC) framework with four domains: agreement and enabling environment, operational standards, quality/efficiency/responsibility, and learning and adaptation. The domains include access, quality of care, financing, community buy in, referral systems, supply and resources of health services [15]. The NOC framework guided site selection, data collection approaches and the initial analytic processes. The project established an expert advisory group (EAG) that included experts in MLBCs from high-, middle- and low-income contexts and representatives of the International Confederation of Midwives (ICM), World Health Organization (WHO), United Nations Population Fund (UNFPA), Bill and Melinda Gates Foundation and World Bank. This EAG met three times during the project to provide advice and guidance.

Prior to commencing the study, human research ethics approval was received through the Alfred Hospital HREC in Australia and from a relevant ethics committee within each of the four countries.

### Country and site selection

Country selection was informed by a literature review and scoping survey, [11, 16] and consultation with ICM and the EAG. The main inclusion criteria were: (a) the country was classed by the World Bank in 2022 as low-, lower-middle, or upper-middle income, (b) there was evidence from the literature and the survey that the country had at least four MLBCs that were either in the public sector or well-integrated within the national health system, (c) good research capacity within the country, and (d) data was available for an economic analysis (to be reported separately). Each country was invited to participate through the national Ministry of Health (MoH) with the ICM member association (MA).

Four study sites were selected in each country based on a desk review of the literature and in consultation with the MoH, the MA, the national research team, the site manager(s) and other relevant stakeholders (Table 1). To be counted as an MLBC, a facility had to be a dedicated space providing childbirth care where midwives were the lead professionals providing care. The midwives were those recognised in each country as midwives. The MLBCs may also have other staff including nurses, lay health workers, assistants-in-midwifery, laboratory technician and pharmacy staff.

**Table 1:** Case study site characteristics

Country	Site no	Location	Rural/ Urban location	Sector	Type
Bangladesh	1	Rajshahi	Rural (Township)	Public	Onsite/alongside
	2	Sylhet	Rural	Public	Freestanding
	3	Sylhet	Rural	Public–private partnership	Freestanding
	4	Chittagong	Rural	Private	Freestanding
Pakistan	1	Punjab	Urban	Private	Freestanding
	2	Sindh	Urban	Private	Onsite/alongside
	3	Balochistan	Rural	Private	Freestanding
	4	Sindh	Rural	Public–private partnership	Freestanding
South Africa	1	Western Cape	Urban	Public	Freestanding
	2	Western Cape	Rural	Public	Freestanding
	3	Eastern Cape	Peri- urban	Public	Onsite/alongside
	4	Gauteng	Urban	Private	Freestanding
Uganda	1	Central	Rural	Private	Freestanding
	2	Central	Rural	Private	Freestanding
	3	Northern	Rural	Private	Freestanding
	4	Eastern	Rural	Private	Freestanding

‘Private sector’ includes for-profit, not-for-profit, NGO. ‘Freestanding’ means a site completely separate from a hospital or other type of health facility to which complicated cases may be referred. Several of the freestanding MLBCs were on the same site as another health facility (e.g. a primary health centre), but not on the same site as a referral facility. ‘Onsite/alongside’ means on the same site as a hospital or other type of health facility to which complicated cases may be referred. The urban/rural classification was as defined by the national research teams

### Study participants, recruitment and data collection

Three groups of informants were purposively selected in each country: health service leaders (defined as midwives, doctors, policymakers, or program planners who, within the preceding two years, had held a leadership role within the maternity care system at a national or sub-national level), MLBC staff (92% of whom were midwives) currently working at one of the selected MLBCs (Table S1: **Definitions of a midwife by country participating in this study**), and MLBC clients (women aged 18 or over who had planned to give birth in one of the selected MLBCs within the preceding six months, including those who had been transferred to another facility). Leaders were selected in consultation with the MoH and the ICM MA. Staff and clients were nominated by the MLBC managers.

Leaders, staff and clients were interviewed separately. Staff were interviewed in focus group discussions (FGDs), or individually if the MLBC was operated by a single midwife. All interviews with clients and staff were conducted in the most appropriate language for that informant or site. The leader interviews were conducted in the informant’s preferred language. FGDs were conducted in a private location at or close to the MLBC. The FGDs lasted between 60 and 90 min and the individual interviews between 30 and 60 min. The majority of the interviews (105/125; 84%) were conducted in person, with the others on the phone or online using Zoom or MS Teams. All interviews were audio-recorded and transcribed in the original language before being translated into English for analysis. The researchers recorded field notes during and after gathering data in order to document the nonverbal cues and significant details observed from the respondents. The study was conducted from September 2022- April 2023.

A total of 210 informants participated: 34–66 per country (Table 2).

**Table 2:** Number of informants by group and country

Participants	Bangladesh	Pakistan	South Africa	Uganda	Total
Health service leaders (interviews)	10	6	9	8	33
MLBC clients (interviews)	21	20	28	8	77
MLBC staff^a^ (focus groups)	28 (4 FGDs)	25 (4 FGDs)	29 (4 FGDs)	18 (3 FGDs + 1 interview)	100 (15 FGDs + 1 interview)
Total	59	51	66	34	210

^a^MLBC staff: 88 Midwives, 4 Nurse-Midwives, 1 Nurse, 1 Lay health worker, 3 assistants to midwives,1 Lab technician, 1 dispenser, 1 pharmacy store keeper

Data collection tools were developed by the research team based on a NOC framework [15] and guided by the Appreciative Inquiry approach [14].  We did not provide a definition of “successful” for the study participants. Rather, we asked them to describe and explore what was working well; and explored what was valued by the participants, including their vision of the ‘ideal’ situation. These aspects of the Appreciative Inquiry approach provided evidence for what makes a ‘successful’ MLBC from the perspectives of the three participant types.

The country research team had a virtual orientation training with international consultants on data collection using the Appreciative Inquiry approach and the team in each country consisted of members with expertise in qualitative research methodology, maternal health and health care. There was a team of 20 researchers in the countries who undertook the data collection. Of these, 15 were women, and 5 were men and all were nationals in the individual country.

### Data analysis

Data analysis was undertaken concurrently with data collection and was initiated after the completion of the first interviews. The translated transcripts were read by a team of four analysts (all women: OB, KH, SCL and ST) who used NVivo software to help organize the data for further analysis. Data were initially categorized under the 4 overarching themes represented in the NOC framework: 1) agreement and enabling environment, 2) operational standards, 3) quality, efficiency and responsibility, 4) learning and adaptation [15]. The next step interrogated the initial analysis asking: (i) What are the enablers, i.e. factors that make MLBCs successful (as defined by individual informants)? (ii) What are the main challenges facing MLBCs? (iii) Do these enablers and challenges vary according to MLBC type (freestanding vs alongside/onsite) and sector (private vs public)? We identified the themes from each of these questions by country and by participant group using an inductive approach and then searched for commonalities and differences. These findings were presented back to the country teams who had collected the data to ensure that the interpretation was sound. Revisions were made and further cross-checking and refinement took place. We then combined the findings across the matrix of country and participant group to produce the synthesized results.

The results are presented initially by context and then what is needed for a successful MLBC. The wider analysis also included the key barriers facing MLBCs and differences between private/public and freestanding/alongside MLBCs (Table S2: **Barriers facing by MLBCs and key differences between different types of MLBCs**).

## Results

### Context

The four selected countries have different strengths and challenges in relation to maternal and newborn health indicators as indicated by Table S3 (Table S3** Key maternal and newborn health statistics for the four countries**).

### What is needed for a successful MLBC?

**Fig. 1:**
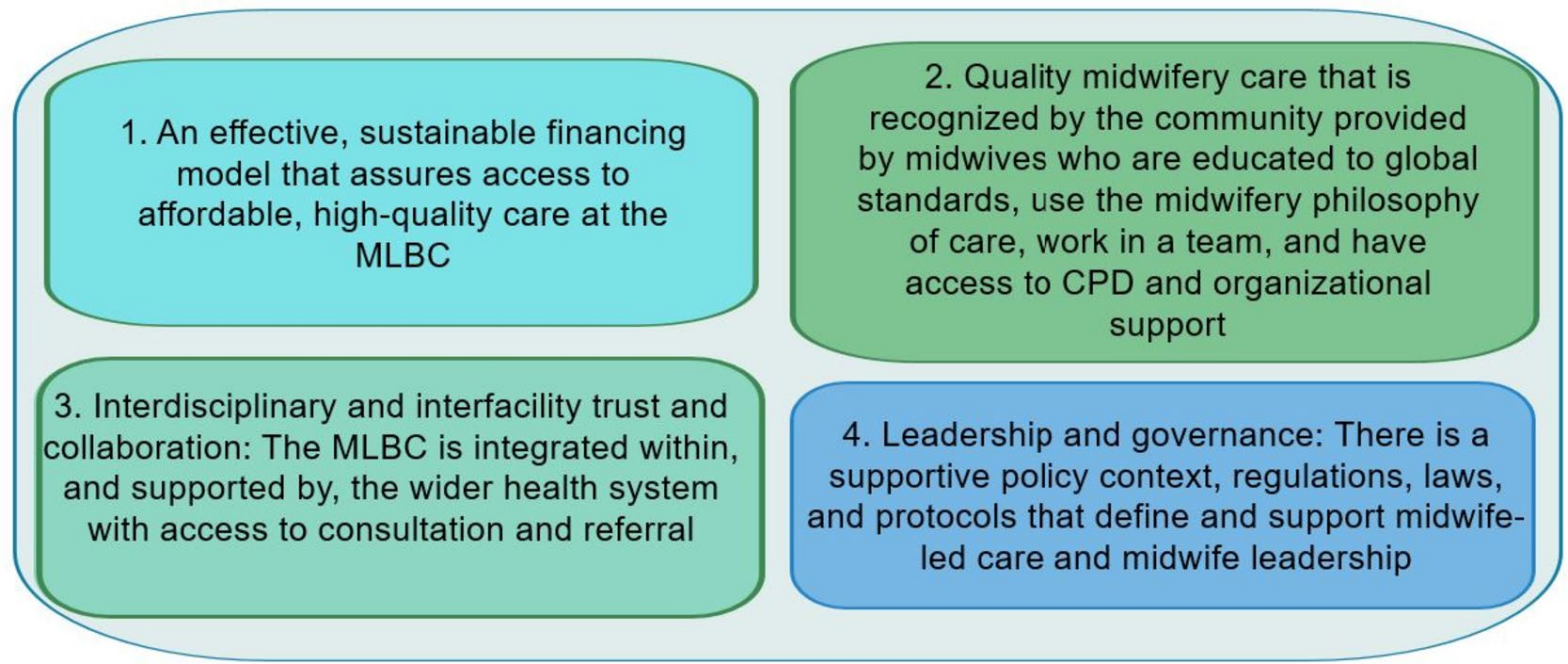
Facilitators of success for midwife-led birthing centres

Although the findings varied by country, four universal themes described the enabling factors influencing successful MLBCs as illustrated by Fig. 1. These were: (1) an effective financing model (2) quality midwifery care that is recognised by the community (3) interdisciplinary and interfacility collaboration, coordination and functional referral systems, and (4) supportive and enabling leadership and governance at all levels.

#### An effective financing model

A number of different financing models for MLBCs services were identified. These included external funding model (non-governmental organizations (NGOs), donors, faith-based organizations, among others), governmental funding model and a combination of two or more funding models. All four countries had external funding models with funders including UN agencies and NGOs, but few South African MLBCs have external funding. Partnerships with these external funders helped to make childbirth care affordable at MLBCs and provided support through donations, as seen in Bangladesh, Pakistan and Uganda. These partnerships improved service utilisation as seen in Bangladesh:

*“Mostly people from the neighbourhood areas come here. They only need to pay for transportation … several NGOs are providing incentives. In that case, a mother is given BDT 500 for a normal delivery. The medication is available here ... It doesn’t cost much. Incentives are supported by UNFPA and [specific programmes], sometimes helps with referrals.”* (Staff Bangladesh)

Sometimes, however, the support was limited and staff had to use their own funds:

*“NGOs provided us with a painted delivery room … I spent my own money to renovate windows, walls, and doors. They [NGOs] do not understand that we are working without any facilities on a low income.”* (Staff Pakistan)

In South Africa, public sector health care included public MLBCs, where pregnant women and children under the age of 6 years are entitled to free health care at the point of care. Government support was also observed in the public health sector in Bangladesh and Pakistan where the government provides infrastructure for MLBCs and the public MLBCs offer free healthcare services. Free services promoted accessibility:

*“I will come here again. I like it here; their way of communicating is very good. I didn’t have to pay anything here; everything is done here for free ... Since it is free and the government pays the expenses here, my relatives will come here happily.”* (User Bangladesh)

*“Mostly our clients are poor and can’t access private services. So they come here… normal delivery is easily done here, and every medicine is free”* (Staff Pakistan).

In Uganda, where the selected MLBCs are all private (some for-profit and some not for profit), priority is given to providing health care to the service user, with payment being made later or some initiative to substitute the cost of care, for example, work in exchange. Two staff members explained:

“*… you do not know if she has money or if she doesn’t have money. So you have to first deliver then after she can tell you “I will send the money”.”* (Staff Uganda)

*“We ask that people either give about five thousand shillings if they can but it is not required or that they can we have some work exchange. People can come and work for a bit and do digging or different things.”* (Staff Uganda)

Different financial models according to the context of the country and flexibility were important to ensure that service users have access to necessary health care without facing financial hardship.

#### Quality midwifery care that is recognised by the community

Successful MLBCs were those recognised by the community as providing quality care in a respectful way. Respect and maintenance of confidentiality for women accessing the MLBC and being treated with dignity and compassion were all important. Midwives in the MLBCs supported physiological labour and birth, catering to the women’s cultural and language needs, establishing strong relationships and ensuring continuity of care. Quality midwifery care included being provided with accurate and unbiased information about their options, being enabled to make informed decisions about their care, and being supported in their choices. Service users explained:

*“…someone understands that you are in pain and they comfort you knowing that if she distances herself from you, you can even die. That love that she is there with you at every point, taking care of you and when you say that I have failed here she is there with you. When you need something she is there with you 24/7. She cares about you and wants your baby to be born well, that nothing happens but for the wellbeing of you and your baby.”* (User Uganda)

*“She [the midwife] never scolded me or anything like that. She treated me with love and respect. She always answered any question I had. She gave me reassurance and told me that I can call for her whenever I need help.”* (User Pakistan)

The examples from Bangladesh and South Africa highlight the value that women placed on receiving care that was respectful, supported physiological labour and birth and reduced the risk of unnecessary interventions:

*“The service providers helped me by respecting my decision to have a normal delivery. I may need something that tells them what they need to look at carefully. In terms of language, there was no difference between them, whether they were poor or rich. Everyone is treated equally.”* (User Bangladesh)

*“I wanted proper care for me and my child. I knew early that I wanted to give birth naturally and I knew that my chances for natural birth would be more reduced when going to a doctor [at a hospital] than going to a midwife [at an MLBC].”* (User South Africa).

This support of physiology was not at the expense of a healthy baby: “…*She [the midwife] cares about you and wants your baby to be born well, that nothing happens but for the wellbeing of you and your baby”* (User Uganda).

Midwives in MLBCs ensured that women felt welcomed and supported. The staff and leaders recognised that this built trust and was important:

*“Patients trust this [MLBC] as we provide good service, take care of their privacy and their self-respect as well.”* (Staff Pakistan)

*“First of all, respect for maternity care is encouraging for [MLBCs]. …When mothers come to these places and see that they can open up, get respect, and give birth the way they want, they gain confidence.”* (Leader Bangladesh)

In South Africa, the involvement of partners and family members during childbirth was highly valued by some MLBC users. They said that midwives accommodated family members and this partner involvement increased awareness of the MLBC:

“*They [the MLBC] were very accommodating, because initially I thought that I am going to give birth with my husband there and then my two sisters came along and then they were very accommodating and then they also involved them throughout like from the labour process and then also assistance*…”(User South Africa)

Cultural and linguistic sensitivity were critical factors in the success of MLBCs. For instance, in Pakistan, service users highlighted that midwives acknowledged and respected the cultural values, beliefs, and practices of the communities they served. The midwives encouraged open communication with the women in their care, which allowed for the sharing of cultural practices and beliefs. Many women from Pakistan felt that the midwives in the MLBC respected their privacy, observed *purdah* (female seclusion) requirements, and communicated with them in their preferred language:

*“We are Baravi speakers. She guides me in Baravi language. She was concerned about my privacy [so] there were no male members in her birth station.”* (User Pakistan*).*

*“She took care of our purdah during check-ups and during childbirth as well.”* (User Pakistan*).*

Building a good relationship with MLBC users was important to provide quality midwifery services and the evidence to relationship is built on trust between midwives and community and on word of mouth:

“*I think it's the relationship between the client and the midwife and the respectful and kind care that is provided by the midwife. It also depends on the feedback that you receive from other people like your sister or your relatives and a lot of community’s trust is built in that way. So I think the [MLBCs] are affordable, acceptable and provide clean quality care and then there are the sources of comfort and respect*”. (Leader-Pakistan)

*“What I found very interesting is that the men/partners become the biggest advocates of midwife-led care or birth centres. They are the ones that then tell their friends, listen you know that this is a good place to give birth.” (*Staff South Africa)

Midwives identified that different aspects of quality of care were key to success, including care across the continuum, and providing remote services. Many MLBCs – especially the freestanding ones—also provided antenatal care. For example:

*“ that relationship begins from antenatal … it can begin when we go to the community and we talk about the availability of this facility [the MLBC], so the relationship begins. And then when they come, the way we talk to them, the way when we are examining them, touching them and talking to them as a family, husband and wife … that puts us together.”* (Staff Uganda)

*“The tasks here are the antenatal care (ANC) check-ups of pregnant mothers, ensuring four visits and quality control of the visits. They also provide remote ANC service through mobile. Many who do not come are called and brought for treatment. Also, the check-ups and various investigations at ANC are done in the hospital itself*.” (Staff Bangladesh)

After birth, MLBCs users were also encouraged to attend scheduled postpartum appointments. In some MLBCs, users were provided with cards to contact the MLBC if any complication arises after discharge:

“*They gave us a card while we were going home from here. A contact number was given on that card so that I could contact them if any complications arose.*” (User Bangladesh).

“…*she [the midwife] was already planning to come the following day, but she said if anything concerns you in this next 24 hours you can just give me a call you know I can come to the house.”* (User South Africa).

Respectful care is a key contributor to success but is still a challenge to achieve, especially when there are midwife shortages. In some settings, clients experienced long wait times, inadequate communication, and lack of timely access to necessary care, especially for the referrals which did not always take place in a timely manner. To address this and ensure success, supportive counselling sessions were organised for midwives at MLBCs in South Africa:

*“We used to have a psychologist … because … to be a midwife sometimes can be very draining because they have been long in labour and sometimes you must look after two patients and you don’t have patience all the time because we are also human, you also coming with the baggage so that it came in that the leader must also look at the staff as a person.” (Staff-South Africa)*

A key to a successful MLBC was the recognition that the centre provided quality care. Midwives were seen as integral to this success but needed access to ongoing education to ensure they were up-to-date with knowledge and skills. In service training was described as an essential component of ensuring that midwives at MLBCs have the knowledge, skills and attitudes necessary to provide high-quality care. Medical doctors, UN agencies and NGOs provided training that included refresher training, neonatal resuscitation and ultrasound:

*“Many NGOs are providing training to fill gaps. NGOs include UNICEF, UNFPA. UNICEF provides us with a wide range of logistical support.”* (Leader Bangladesh)

*“In three months, basically we get a refresher on different subjects, its normally about the staff need" [referring to Essential Steps in Managing Obstetric Emergencies, Helping Babies Breathe training].”* (Staff South Africa)

*“We went to Karachi and attended ultrasound training and that was very good. Everyone from here went there one by one from training and that was good. Our knowledge increases from that training.”* (Staff Pakistan)

#### Interdisciplinary and interfacility collaboration, coordination and functional referral systems

Collaboration, coordination and functional referral systems are needed for successful MLBCs. While the MLBC midwives are the lead professionals, collaboration with obstetricians, nurses, and other support staff and working as a wider team were important. “*I would say the common goal that we are having, no mother and child should die. Teamwork is key amongst all of us the midwife fraternity [sic].”* (Leader Uganda). This sense of collaboration and teamwork was also expressed in South Africa and Pakistan:

*“Yes I want to say what makes my day is everyone getting along and more patients giving birth and it is smooth, happy team work day, everyone is smiling, there is no fighting … It can get very hot and steamy in the labour ward, and … I do find that we all have good relationships with each other under those situations and we can still make good decisions now walking away from that knowing that you did the best with what we had.”* (Staff South Africa)

*“…We deal with everything together. ….. Our doctors are also very cooperative.”* (Staff Pakistan).

Good coordination included flexibility in how the service is organised and enabled continuity of care:

*”I liked that idea that even if my midwife was unavailable, there were back up ones that I could meet ahead of time and get to know a little bit about care, yeah just bit more of personal care.”* (User South Africa)

Staff also valued being able to organise and coordinate their own work:

*“That depends on us [how] we have to divide it [the work] shift wise. Sometimes we divide the burden in the shifts so that not all the burden is on the first shift. So we have to make those policies ourselves. We don’t have any pressure from our admin or head office. This is the work we do on our own with their cooperation and there isn’t any more change needed in that.”* (Staff Pakistan)

A well-functioning referral system was critical for successful MLBCs to ensure that, when needed, women received appropriate and timely care at a referral hospital with advanced care. The referral system was facilitated by effective communication and coordination from MLBCs to referral hospitals. Staff and leaders explained:

*“So, from up to down, we have free communication, policies and protocols that determine which patient is where, and they are appropriately referred and referred back again.”* (Leader South Africa)

*“There were identified sites where we would refer the patients and you ring the doctor before you send the patient. So, even if it’s like APH [Antepartum haemorrhage], PPH [Postpartum haemorrhage], now the doctor would say do a,b,c,d as you are coming with this patient and that was really beautiful. So having connections of where to refer, so that by the time the patient reaches there, there is somebody waiting for the patient.”* (Staff Uganda)

*“I like working here in an [MLBC] which is adjacent to the main labour ward because if there is any complication there are those intrapartum emergencies like shoulder dystocia, PPH, uterine inversion, so you are just close to labour ward of the hospital for further management.”* (Staff South Africa)

*“What I liked about this clinic is that they are able to quickly see the problem if you have it, then when they see that it is not theirs and they will not be able to help it, they are able to refer you to higher people who will be able to help you.”* (User South Africa)

Some sites ensured there was feedback about the transfer or systems where staff keep in touch via phone with service users. For example in Bangladesh, leaders found this feedback important in order to ensure that service users get enough support to maintain health:

*“...the patient is then transported by us to another health facility with a midwife. Even after the referral, we keep in touch with the mother every day through her mobile phone.”* (Leader Bangladesh).

#### Supportive and enabling leadership and governance at all levels

Effective leadership and governance systems enabled successful MLBCs. Governance mechanisms in different forms were described and included policy, governmental support and recognition, coordination meetings, support for continues professional development and monitoring and evaluation to ensure the MLBCs function and are effective. Government support to MLBCs was seen as a contributing factor to quality of care and hence reduction in maternal and child mortality rates:

*“The government supports this service led by midwives. That is why the government has created separate midwives for maternal and child health and is providing full cooperation. They are providing as much support as needed. The main objective of the government is to reduce maternal and child mortality rates and increase institutional deliveries. It is for these reasons that the government is supporting midwives.”* (Staff Bangladesh)

*“Well, we get support from the government in the sense that they recognize our practice, and we can transfer patients to [institution names].”* (Staff South Africa)

*“The district is very supportive. You can’t run this type of, you know, activity without support from the district and the community.”* (Staff Uganda)

*“[MLBCs] have developed good setups and made a good network but it all depends on how much support they got. It depends on how your seniors, supervisor, NGO or the government held your hand. So that is very important. Once they are backed up, they can do wonders.”* (Leader Pakistan)

Coordination meetings and regular supervision involving MLBCs brought together stakeholders to discuss improving the quality and accessibility of MLBC services. Coordination meetings enabled MLBC staff, government officials, healthcare providers, community leaders and other stakeholders to share information and experiences and contribute to effective functioning:

*“We have quarterly meetings … to review the performance of each facility. We have also monthly meetings for the in-charges to review our performance and then we also do quarterly supervision and we have tools that guide us that we use to assess the quality-of-care services. Then we also have, they also submit reports to the district and we enter them into our system.”* (Leader Uganda)

*“There was a monthly perinatal meeting and later there was ESMOE [Essential Steps in Managing Obstetric Emergencies]. So, I suppose I then also got to know how they function and then later on [community obstetrician] kind of really coordinated the [MLBCs] in Cape Town and he set up a meeting called MOUSE [Midwife Obstetric Units Support Executive].”* (Leader South Africa)

It was also highlighted by staff in South Africa that availability of guidelines and protocols at the disposal of midwives facilitate in decision-making for transfer. Leaders in Pakistan also had guidelines and frameworks but these were not applied in private MLBCs:

*“We do have written guidelines and protocols in labour ward and in the antenatal clinic that we follow especially when it comes to transfers, what to transfer, so we work according to the protocol.” [Staff South Africa]*

*“In the infrastructure [MLBC], there should be midwifery led policies rather than medical policies. There should be a midwifery led service framework and the policies should be according to it. So that women can get midwifery services rather than medical services.” (Leader Pakistan)*

Effective leadership and governance play a crucial role in enabling the timely and accurate reporting of data, ensuring evidence-based services at MLBCs. The establishment of reliable monitoring and evaluation mechanisms at MLBCs is an essential element for their successful operation and impact.:

*“Evidence is mainly maintained by register books. We maintain it step by step from entry to discharge ... Thus, evidence-based services are ensured. We have been given a register of maternity services from the government. We fill up the form as given there.”* (Leader Bangladesh)

“*In the government centres, the data is present in two ways. Firstly it is present in the paper form where there are separate registers for antenatal patients, delivery and for postnatal. Now in the government centers of Punjab, a new system of registration called EMR has started, which is known as Electronic Medical Reporting. Through it, every patient’s registration is being done through an application.” (Staff Pakistan)*

*“We report to the district every month. They have a tool that they give us but then we also keep our own private records. So we record with, you know, we have our paper charts that we have on each client and then we have someone who takes those paper charts and puts them into a larger book and then we take that book once a month and we digitize it.”* (Staff Uganda)

## Discussion

The need to accelerate progress on maternal and newborn health and wellbeing is widely recognised. Action is required to increase the availability and accessibility of maternal and newborn health care, and also to improve quality of care (including respectful care). Expanding access to MLBCs is one means of achieving these objectives. Prior to this study, most of the evidence about MLBCs came from high-income countries, and the extent to which conclusions based on this evidence were transferable to LMICs was not clear. This study in four LMICs demonstrates that MLBCs are a feasible and acceptable model of care in a wide range of settings. The findings highlight that leadership support, availability of adequate resources, and workforce development as potential factors contributing to the successful implementation of MLBCs in LMICs.

Scaling-up the MLBC model of care to reach more women in more countries requires attention to be paid to the enablers identified in this study. High level themes describing enablers are: (i) having an effective financing model (ii) providing quality midwifery care that is recognised by the community (iii) having interdisciplinary and interfacility collaboration, coordination and functional referral systems, and (iv) ensuring supportive and enabling leadership and governance at all levels. With these enablers in place, the potential for MLBCs to contribute to improved maternal and newborn health and wellbeing is enormous. Our findings are discussed in view of evidence from both LMICs and HICs (high-income countries).

There are strong parallels between the findings of this study and recently published research from LMICs about enablers to the implementation of midwife-led care in LMICs, which highlighted the importance of funding, community accessibility and trust, quality of care (including respectful care), leadership and collaboration [17–20]. Our findings suggest that financing is crucial for the sustainability and accessibility of midwifery care services. When there is a stable and sufficient funding mechanism in place, MLBCs can maintain their operations, invest in necessary resources, and provide quality care to expectant mothers. Adequate financing can also contribute to improving infrastructure, equipment, and training opportunities for midwives, which, in turn, enhances the overall quality of care. Studies have shown that various financing models can support midwife-led care, including government funding, private insurance coverage, and community support. In a scoping review exploring network of care in implementing MLBCs in LMICs, strategies to make MLBC services affordable were identified: such as establishment of a health insurance scheme and incentivised services or fee waivers for maternity services to increase utilization. Communities utilise MLBC services more when the services were either free or subsidised through an enabling environment that included health insurance, incentives, and donor support [16]. 

Our results indicate that community recognition and acceptance of midwifery care are essential for the success of midwife-led birthing centres. When the community values and trusts the expertise of midwives, more women and families will opt for midwifery care during childbirth. This can lead to increased demand for MLBCs, resulting in better outcomes for mothers and babies. Our study also highlights that midwives in the MLBCs support physiological labour and birth, catering to the women’s cultural and language needs, establishing strong relationships and ensuring continuity of care MLBCs. To achieve community recognition, MLBCs need to prioritize providing high-quality, evidence-based care. This includes offering personalized and comprehensive prenatal, intrapartum, and postpartum care to expectant mothers. Additionally, fostering strong communication and relationships between midwives and their clients can contribute to a positive reputation.

Collaboration and coordination among different healthcare professionals and facilities are vital components of a successful MLBC. MLBCs are likely to be successful if they are integrated within the broader health system [18, 21]. The results of our study demonstrate that this does not necessarily mean that the MLBCs must be part of the public sector system; they can be private, NGO-run or a combination. Integration can be achieved across different sectors if the MLBCs are part of a well-functioning network of care. In settings with low capacity in the public sector, it is important to pay attention to how such networks can be built and/or strengthened in order to facilitate the expansion of access to high quality MLBCs.

The sites we included were those that provided care during labour and birth. Most of the included sites also provided other maternal and newborn health care services such as antenatal and postnatal care. Although these other elements of care were not the main focus of this study, evidence from other studies indicates that midwife-led continuity of care models providing care across the continuum have numerous benefits over other models of care [9, 22–26]. Efforts to expand access to high-quality MLBCs should take into consideration the potential benefits of ensuring that care is offered across the full continuum of care where feasible and appropriate.

Our study findings also align with similar studies in HICs [27–30] and the frameworks and standards that have been developed based on those studies [31–33]. For example, the following factors have been identified as important in a range of settings as well as in this study: an enabling policy environment including adequate financial and human resourcing, [28–33] mainstreaming MLBCs as a core element of the service rather than as an ‘optional extra’, [27, 28, 30] strong and inspirational midwifery leadership, [27, 28, 30] education and training programmes that prepare midwives with the competence and confidence to support physiological birth and make decisions independently, [28, 30] interdisciplinary and interfacility collaboration built on relationships of trust and respect [28, 30]. Supportive leadership helps create a positive work environment for midwives, encouraging their professional development and job satisfaction. It also ensures that the MLBC operates efficiently, adheres to best practices, and maintains a focus on providing quality care.

The findings of this study have significant implications for improving maternal and neonatal health outcomes, strengthening healthcare systems, and promoting the role of midwives in LMICs. We have identified key factors that enable the successful establishment and operation of MLBCs. Understanding these factors can contribute to inform policies and decision making as well as design tailored maternal and newborn health programmes that can more effectively support midwives and respond to population needs. At an international level, it can contribute to shape guidelines and strengthen the midwifery profession in different settings.

### Strengths and limitations

One of the strengths of our study is its approach to triangulation used to obtain credible information on enablers for the establishment and running of MLBCs in LMICs. The use of multiple data collection methods (KIIs and FGDs) with different groups of informants – including women who have used MLBC services—to corroborate findings increased the validity and reliability of the study's findings. The involvement of a team of four data analysts and the data validation from the four country teams was also a strength to ensure rigour in data analysis and increase the credibility of the study. However, there was no systematic attempt to conduct repeat interviews or ask participants to validate the interview transcripts, which may have adversely affected the richness of the data.

Methodological limitations occur often in qualitative study involving multiple sites in multiple countries with different cultural contexts and languages. Differences in the interpretation of questions or cultural norms may affect the quality and consistency of the data collected. To counteract this, each country team of researchers underwent training on the research approach and methods used. The country teams identified experienced translators fluent in the appropriate languages to translate both interview guides and transcripts.

We included MLBCs that had midwives as recognised in their country. We acknowledge that there are a range of midwives including community midwives and nurse-midwives and not all may meet the definition of a midwife according to the ICM [34]. This is likely in many countries who are transitioning to having midwives educated according to ICM standards, but it will take some countries many years to ensure all their MLBC staff fit this definition. Despite this, these health workers were working in the capacity of midwives providing care within a midwifery model of care and it is very encouraging to see the positive impact they were having in their communities.

We did not systematically include the perspective of other leaders including midwives' associations and regulatory bodies who may have a crucial role in promoting the safety and quality of care in MLBCs. Therefore, future research should explore the perspectives and experiences of midwives' associations and regulatory bodies in supporting MLBCs. This will provide a more comprehensive understanding of the factors that contribute to the success of MLBCs and inform policy and practice in this area.

## Conclusion

The aim of this study was to explore MLBCs in four low- and middle-income countries, specifically to understand what is needed for a successful MLBC. We have shown that the key elements of success include having an effective financing model; providing quality midwifery care that is recognised by the community; having interdisciplinary collaboration, coordination and functional referral systems, and ensuring supportive and enabling leadership and governance at all levels. MLBCs are one way to deliver midwifery interventions which have been shown to reduce maternal and newborn deaths and stillbirths. Understanding factors for success can contribute to inform policies and decision making as well as design tailored maternal and newborn health programmes that can more effectively support midwives and respond to population needs. The next step is to scale up MLBCs while collecting evidence on the effectiveness of this model of care on the outcomes for women, babies, midwives and the health system.

### Supplementary Information

Additional file 1: Table S1. **Definition of a midwife by countries included in the study**.

Additional file 2: Table S2. **Barriers facing by MLBCs and key differences between different types of MLBCs**.

Additional file 3: Table S3. ** Key maternal and newborn health statistics for the four countries**.

**Additional file 4:** **Supplementary 4. **Interview guiding questions.

## Data Availability

The datasets generated and analysed during the current study are not publicly available due to the risk of compromising participants’ confidentiality, but are available from the corresponding author on reasonable request.
