# Supplementary material for: Midwife-led birthing centres in four countries: a case study
Source: BMC Health Serv Res. 2023 Oct 17;23:1105. doi: 10.1186/s12913-023-10125-2 (PMC10583445; doi:10.1186/s12913-023-10125-2)
Supplement: Supplementary file 1 — Additional file 1: Table S1. **Definition of a midwife by countries included in the study**. [file 12913_2023_10125_MOESM1_ESM.docx]

**Table S1: **Definition of a midwife by countries included in the study****

| **Country** | **Definition** | **Source** |
| --- | --- | --- |
| Bangladesh | The midwife in Bangladesh is a qualified (diploma or degree) and registered trained professional someone who has completed a recognized educational programme (three years diploma course or 4 years BSc course) in midwifery and is licensed by the Bangladesh Nursing and Midwifery Council as a registered midwife. She would work in partnership with the woman and her family throughout the maternity experience.  The Bangladesh Nursing and Midwifery Council and Bangladesh Midwifery Society accept the International Confederation of Midwives (ICM) definition of a midwife (2011): “A midwife is a person who has successfully completed a midwifery education programme that is duly recognized in the country where it is located and that is based on the ICM Essential Competencies for Basic Midwifery Practice and the framework of the ICM Global Standards for Midwifery Education; who has acquired the requisite qualifications to be registered and/or legally licensed to practice midwifery and use the title ‘midwife’; and who demonstrates competency in the practice of midwifery. | Bangladesh Nursing and Midwifery Council (BNMC) and BNMC Act 2016 |
| Pakistan | “A midwife is a person who has successfully completed a midwifery education program (Approved by Pakistan Nursing Council) that is based on the ICM Essential Competencies for Basic Midwifery Practice and the framework of the ICM Global Standards for Midwifery Education and is recognized in the Pakistan; who has acquired the requisite qualifications to be registered and/or legally licensed to practice midwifery and use the title “midwife”; and who demonstrates competency in the practice of midwifery.” Adapted from ICM 2017 | Pakistan Nursing Council and the Curriculum of Midwifery Education BSM (Baccalaureate studies of Midwifery) (2021) |
| South Africa | “A midwife is a licensed person who is registered with the South African Nursing Council (SANC) based on the completion of a recognized education and training programme to nurture, assist and treat the client, who can be a woman, a neonate or a family, in the process of promoting a health pregnancy, labour and postpartum period. In working with the clients according to prescribed professional codes, they acknowledge them as equal partners” (SANC, 2021) | South African Nursing Council, 2021, Circular 5/2001 Scope of practice of the Nurse and Midwife. <https://www.sanc.co.za/2>001/05/16/circular-5-2001-scope-of-practice-of-the-nurse-and-midwife/ |
| Uganda | “A midwife is a person having been, admitted to a midwifery education program has successfully completed the prescribed course of studies, acquired the necessary qualifications and is duly recognised by the appropriate regulatory body, registered and licensed to practice midwifery” | The Republic of Uganda ,2017, Ministry of Public Service. Schemes of service for the nursing and midwifery care, www. publicservice.go.ug. |
