# Supplementary material for: Midwife-led birthing centres in four countries: a case study
Source: BMC Health Serv Res. 2023 Oct 17;23:1105. doi: 10.1186/s12913-023-10125-2 (PMC10583445; doi:10.1186/s12913-023-10125-2)
Supplement: Supplementary file 2 — Additional file 2: Table S2. **Barriers facing by MLBCs and key differences between different types of MLBCs**. [file 12913_2023_10125_MOESM2_ESM.docx]

**Table S2: **Barriers facing by MLBCs and key differences between different types of MLBCs****

**What are the key barriers facing MLBCs?**

| **High level themes** | **Description** | **Sample quote for illustration** |
| --- | --- | --- |
| Funding models that reduce affordability | Ineffective funding models created challenges for women accessing the MLBC and midwives providing the service.  The lack of a ‘ring-fenced’ budget or protected funding for the MLBC limited their capacity to operative effectively.  Indirect costs to the users, like the need to pay for transport to access the MLBCs, impacted access and meant women went elsewhere for care | *“So you need to first of all have funds before you even initiate a maternity centre so that you are able to pay, because you have to pay the workers. You can pay, you are able to have the resources, the supplies, the drugs and the people will come but if you initiate it without funds then you are most likely not to get the standards for human resource”* (Leader Uganda) |
| Inadequate infrastructure and equipment, which compromises safety and quality | Inadequate facilities compromise the safety and quality of care provided to mothers and their newborns  Limited space in the MLBC and access to essential infrastructure  Limited funds meant inaccessibility of full midwifery care at MLBC especially when there were inadequate supplies of essential medicines  Geographical accessibility, particularly in hard-to-reach communities, was a challenge across the different study sites | “*We travel here by auto rickshaws and rickshaws. I arrived by auto while delivery had already started on my way to the centre. As I was healthy, it was fine. But that wouldn’t be right if I was seriously sick. The transportation system should be improved to easy travel to here”* (User Bangladesh) |
| Limited support to / trust in midwives | Shortage of midwives, low salaries, a lack of professional recognition of midwives, limited skills to manage maternal complications and lack of specific guidelines for independent midwife-led care. | *“Unfortunately, sometimes you have skilled trained staff leaving one MLBC to other areas because they feel that they are not well compensated for the skills that they do have. You may also find a highly skilled person serving an administrative post because they feel that they are not remunerated for the level of services that they render and I think that’s where we can possibly make a difference to make sure that highly skilled and trained staff are adequately compensated in order to keep them there* (Leader South Africa) |
| Poor integration of MLBCs within the health system | Limited health insurance coverage,  Lack of coordination of referrals and continuity of care  Lack of governmental support | “*Referral is usually not very formalized. It is developed by the Staff through their relationship and communication. These are almost informal mechanisms and not formal mechanisms and that’s why the care provision is not that good”* (Leader Pakistan) |

**Key differences between private/public and freestanding/alongside MLBCs**

| **Public MLBCs** | **Private MLBCs** |
| --- | --- |
| More likely to be integrated into the health system | Less well integrated into the health system, except in Uganda |
| More sustainable financing mechanisms: Governmental support and free care | Both private for-profit and non-profit MLBCs required user fees.  Donor-financed MLBCs/ Non-profit MLBCs appear to be well equipped and suffer fewer stock-outs of medicines. For-profit MLBCs seem more likely to suffer from a lack of equipment and poor facilities. |
| ***Alongside/onsite MLBCs*** | ***Freestanding MLBCs*** |
| Respectful care was a strong theme across all types of MLBCs | Appreciation for respectful care was particularly noticeable in freestanding, independent practices |
| Midwives working in larger alongside/onsite MLBCs hospitals valued good teamwork and good relationships with other health workers including doctors. | Midwives running freestanding independent private practices often lacked collaboration with other midwives or doctors and shouldered more responsibility, including the training of their assistants. |
