# Supplementary material for: Midwife-led birthing centres in four countries: a case study
Source: BMC Health Serv Res. 2023 Oct 17;23:1105. doi: 10.1186/s12913-023-10125-2 (PMC10583445; doi:10.1186/s12913-023-10125-2)
Supplement: Supplementary file 3 — Additional file 3: Table S3. ** Key maternal and newborn health statistics for the four countries**. [file 12913_2023_10125_MOESM3_ESM.docx]

**Table S3** Key maternal and newborn health statistics for the four countries****

|  | **Bangladesh** | **Pakistan** | **South Africa** | **Uganda** |
| --- | --- | --- | --- | --- |
| Maternal mortality ratio 2020 [16] | 123 | 154 | 127 | 284 |
| % change in maternal mortality ratio 2000-2020 [16] | -72% | -62% | -25% | -39% |
| Neonatal mortality rate 2021 [17] | 16 | 39 | 11 | 19 |
| Stillbirth rate 2021 [18] | 21 | 31 | 16 | 15 |
| Total fertility rate 2022 [19] | 1.9 | 3.3 | 2.3 | 4.5 |
| Number of live births (millions) 2023 [20] | 3.0 | 6.5 | 1.1 | 1.7 |
| % of live births attended by skilled health personnel [21] | 59% (2019) | 68% (2020) | 97% (2016) | 74% (2016) |
| % of live births in a health facility [21] | 53% (2019) | 70% (2020) | 96% (2016) | 73% (2016) |
| Caesarean section rate [22] | 33% (2018) | 22% (2018) | 24% (2016) | 6% (2016) |
| % of pregnant women accessing 4 or more antenatal care visits [21] | 37% (2019) | 52% (2019) | 76% (2016) | 57% (2018) |
| % of postpartum women receiving a postnatal check within 2 days of birth [21] | 65% (2019) | 69% (2019) | 84% (2016) | 54% (2016) |
| % of newborns receiving a postnatal check within 2 days of birth [21] | 67% (2019) | 64% (2018) | 86% (2016) | 56% (2016) |
| Midwifery professionals per 10,000 population [23] | 0.3 | 0.7 | 0.2 | 2.2 |
| Midwifery associate professionals per 10,000 population [23] | 0.4 | - | - | - |
| Potential met need for sexual, reproductive, maternal, newborn and adolescent health care [23] | 57% | 61% | 56% | 50% |
| National policy for midwife-led care in childbirth? [23] | Yes | Yes | Yes | Yes |
| National policy on midwife education based on ICM competencies? [23] | Yes | No | No | Yes |
| National policy on midwife regulation based on ICM competencies? [23] | Yes | No | No | Yes |
| Is continuing professional development a requirement for midwife relicensing? [23] | No | - | - | Yes |

Sources: shown separately for each indicator
