# Supplementary material for: Midwife-led birthing centres in four countries: a case study
Source: BMC Health Serv Res. 2023 Oct 17;23:1105. doi: 10.1186/s12913-023-10125-2 (PMC10583445; doi:10.1186/s12913-023-10125-2)
Supplement: Supplementary file 4 — Additional file 4: Supplementary 4. Interview guiding questions. [file 12913_2023_10125_MOESM4_ESM.docx]

**Supplementary 4: Interview guiding questions**

**A. Health care leaders and policy makers**

**Guiding questions in key informant interviews with health service leaders and policy makers**

*Notes for interviewer: Please thank the interviewee for participating in this interview. Please confirm that the person has read the Information Sheet and has signed the consent form.*

*Please remind the interviewee that the audio recording will now commence prior to the interview.*

***Introductory questions:***

- Explore how the respondent’s organisation is involved in MLBCs, the respondent’s own role
- Describe the history of MLBCs in the country: when they were established, why and by whom
- Describe the different types of MLBC that exist in the country (probe for: location, services offered in addition to childbirth, physical design/layout, public or private sector, how they are funded, who can access care at an MLBC, what services are provided in addition to childbirth care)
- How do MLBCs fit within the wider health system?

***Key interview questions:***

- How does the government support the MLBCs? (What is the position of government in relation to MLBCs?)
- Which other organisations (e.g. NGOs) support the MLBCs and in what ways?
- How is the community engaged or involved? (How do you get buy-in from the community?)
- What factors encourage women to trust the MLBC services?
- How are MLBC services made affordable for the users? (What is the main payment method for service users? What financial support does the government provide for service users?)
- How do the MLBC services fulfil the community’s needs? (probe for social and cultural needs as well as clinical needs)
- What would it look like if they were to meet the community’s needs fully?
- What are three main things that need to happen to make the services more affordable, acceptable and fit for purpose?

- How does the referral system work? What happens when this functions well?
- How are referral pathways integrated within the health system? What would an ideal referral system look like? (How is the information communicated and shared with all levels of care? How are service users told about the referral system?)
- What data management systems do you have and how could these be strengthened?
- How are supplies and equipment for providing high quality services at MLBCs ensured? What does the supply system look like when supplies and resources are fully provided?
- Describe the ideal staffing levels for MLBCs (workforce numbers and cadres)?  What needs to happen to make this a reality?
- Who is in charge of the MLBCs? (If midwives or nurse-midwives, ask for more information about which cadres of midwife or nurse-midwife and how they are different from any other type of midwife that exists in the country) What does being “in charge” look like? (Are the in-charges autonomous, or do they report to a higher authority?)
- What are three things you would change in terms of workforce and infrastructure?
- What factors facilitate effective coordination and partnership between MLBCs and other types of health facility? Please give examples. How could this be improved?
- What factors facilitate effective coordination and partnership between midwives in MLBCs and other health professionals involved in maternal and newborn health care? Please give examples. How could this be improved?
- What competencies should providers have to work within an MLBC? What needs to happen to make this a reality?
- In what ways are the services provided within MLBCs evidence-based? (What guidelines or standard operating procedures exist (if any), and do all providers follow the guidelines?) How do you know?
- What does high quality care in MLBCs look like? What factors facilitate high quality care in MLBCs?
- How is the quality of care assessed and evaluated?  (probe for quality of data and data management systems)
- If you were planning to improve the quality and efficiency of MLBC services, what would be the three main things you would do?
- What 3 aspects of MLBCs in this country are you most proud of and why? (Probe: Is there something innovative and unique that is provided as part of MLBC services, and other facilities may not deliver that? What are you most proud of?)
- Apart from regular services you are providing, do MLBCs serve communities who might otherwise not be able to access care?
- What modern approaches or technology are used as part of MLBC services? Please give examples of how these have been useful/valuable.

**B. Health care workers**

**Guiding questions in Focus group discussions with Health workers**

*Notes for focus group facilitator: Please thank the participants for participating in this focus group. Please confirm that the participants in the group have read the Information Sheet and have signed the consent form.*

*Please remind the participants that the audio recording will now commence prior to the focus group.*

**Introductory questions:**

- Explore the respondent’s role in the MLBC, their gender, how long they have worked there, was it their own choice to work there or were they just deployed there?
- Describe your colleagues at the MLBC - who else works with you, how do their roles and responsibilities compare to yours, who is in charge (and what does “being in charge” look like)?

***Key questions for focus group discussion:***

- How does the government support MLBCs? How do other organisations (e.g. NGOs) support MLBCs?
- How do you engage or involve the community? (How do you get buy-in from the community?)
- What does an ideal relationship between an MLBC midwife and her client look like?
- If you were a pregnant woman, what factors would make you trust the MLBC services?  (Why?)
- How do you address the specific needs of the community? (Are there any barriers or enablers?)  What would you do to make the services more acceptable by the community?
- What is the main payment method for service users? Which women can afford MLBC services? Does the government or other organisations arrange any financial support for the service users? (explore in detail)

- How do you ensure that the MLBC has all the supplies, equipment and resources it needs to provide high quality services?
- What kind of information do you record about MLBC performance? (probe for central data management  system and if they comply with data collection requirements)
- How do you manage emergency/ complicated cases? (Probe: Where do you refer the client? What kind of agreement with other health facilities do you have? How do they get there?) What does it look like when the referral system works well?
- What are the best things/moments for you as a provider working in MLBC? (What do you enjoy the most?) How do you feel about working within MLBC? What would you like to change in future to make the situation better for you? (probe for burnout, supports and workload)
- What competencies do providers need to work in an MLBC? Do you think all health care providers in this MLBC have all the required competencies? If not what should be done to improve it?
- How do you ensure that the care that you provide is evidence-based? What main guidelines and standards do you use? Are those guidelines helpful? What else needs to be done?
- If you were planning to improve the quality and efficiency of MLBC services, what would be the three main things you would suggest?
- In what ways is the care provided client/women-centred?
- Is there something innovative and unique that your MLBC provides and that facilities do not? (Apart from regular services you are providing) How does this benefit the clients and/or the health care providers?
- Is there any modern approach or technology that you are using as part of MLBC services? How does this benefit the clients and/or the health care providers? What kind of technology or approaches would improve services in future?
- How do you facilitate access to MLBC services for those who might find it more difficult to access care here, e.g. because they are poor or cannot easily travel? How could you improve this in future?

**C. Women**

**Guiding questions in interviews with women accessing MLBCs**

*Notes for interviewer: Please thank the interviewee for participating in this interview. Please confirm that the person has read the Information Sheet and has signed the consent form.*

*Please remind the interviewee that the audio recording will now commence prior to the interview.*

***Introductory questions***:

- Tell me about your most recent birth at (name of MLBC).
- When was it? Did you have a son or a daughter?
- Was it your first birth? If not, where did you give birth before?

***Key interview questions:***

- How did you hear about the MLBC and why did you choose it?
- What did you like about the MLBC?
- What did you like about the staff of the MLBCs? (Did you feel comfortable to share things or ask questions?)
- How did they involve you and your family in decisions about your care?
- In what ways did the MLBC respect your needs? (probe for things like: birth partners, language, respect for cultural traditions that are important to the woman)
- What or who helped you to pay the costs of accessing care? (probe as appropriate for: user fees, transport costs, food and accommodation for self and family members, medicine costs, equipment costs (e.g. sanitary pads))

- Do you think the MLBC has all the health workers, materials and equipment it needs to provide high quality childbirth services? What should be done to make it better in future?
- What did the midwives do to make you feel confident that they knew how to do their job well?
- What did the midwives do to make you feel confident in your own ability to give birth safely and care for your baby?
- What documentation and paperwork did they give you when you were discharged from the MLBC?
- Before you gave birth, what information did the MLBC give you about what would happen if there was a complication or emergency that meant you needed to transfer to a hospital?
- Did you or your baby need to be transferred to another facility either during labour or shortly after the birth? Why? Tell me about that experience. How did you feel?

- How did you make the journey from your home to the MLBC? What would have made that journey easier for you?
- What is it about the MLBC that makes it different from other health facilities where women can give birth?
- How did the midwives make you feel respected?
- How did the midwives encourage you to ask questions and ask for what you needed?
- How did the midwives encourage you to make your own decisions about your care?
- Would you give birth at MLBC again in future? Would you recommend the MLBC services to other women? Why?
- What are three main things you would like to see changed for better services in future?
